# Comment on ‘Muscle‐Specific Strength Better Predicts Physical Performance Decline Than Conventional Metrics: The I‐Lan Longitudinal Aging Study’ by Chien et al.—The Authors Reply

**DOI:** 10.1002/jcsm.70238

**Published:** 2026-02-18

**Authors:** Wei‐Ju Lee, Wen‐Kai Chien, Liang‐Kung Chen

**Affiliations:** ^1^ Institute of Hospital and Health Care Administration National Yang Ming Chiao Tung University Taipei Taiwan; ^2^ Center for Healthy Longevity and Aging Sciences National Yang Ming Chiao Tung University Taipei Taiwan; ^3^ Department of Family Medicine Taipei Veterans General Hospital Yuanshan Branch Yi‐Lan County Taiwan; ^4^ Center for Geriatrics and Gerontology Taipei Veterans General Hospital Taipei Taiwan; ^5^ Taipei Municipal Gan‐Dau Hospital Taipei Taiwan

We thank Dr. Lima and Dr. Blazevich for their insightful comments on our recently published work, “Muscle‐Specific Strength Better Predicts Physical Performance Decline Than Conventional Metrics: The I‐Lan Longitudinal Aging Study.” [1] Their observations regarding the paradoxical characteristics of individuals with low muscle‐specific strength (MSS), the influence of adiposity on strength interpretation and the potential role of relative handgrip strength (rHGS) raise important considerations for advancing the assessment of muscle function.

As the commenters noted, participants with low MSS have higher skeletal muscle index despite lower absolute grip strength, suggesting a phenotype marked by elevated adiposity and reduced contractile efficiency. They further proposed that grip strength normalized to body mass may better capture functional demands during daily activities, consistent with evidence linking rHGS to cardiometabolic and physical outcomes [2, 3]. These perspectives align with evolving consensus in the field. The Asian Working Group for Sarcopenia (AWGS) 2025 Consensus supports the concept that muscle‐specific strength is emerging as a more sensitive indicator of muscle function than absolute strength or muscle mass alone [4]. Similarly, the Global Leadership Initiative on Sarcopenia (GLIS) recommends the use of either muscle strength or muscle‐specific strength as the primary functional component in sarcopenia assessment, while acknowledging that MSS remains under methodological refinement and requires further empirical validation [5]. Given that individuals with low MSS in our cohort also demonstrated higher adiposity, these observations are highly relevant to growing discussions about muscle quality, efficiency and sarcopenic obesity.

To address the scientific questions raised, we conducted supplemental analyses within the ILAS cohort comparing MSS with three forms of rHGS—normalized to body weight, body mass index (BMI) or height^2^—in predicting physical decline [3]. This analysis used the same participants and baseline measurements as described in our original publication [1]. MSS was calculated as handgrip strength divided by dominant‐hand lean mass, with low MSS defined as the lowest age‐ and sex‐specific quintile. In parallel, rHGS metrics were constructed using the same quintile‐based classification. Although the commenters suggested applying The Sarcopenia Definition and Outcomes Consortium (SDOC) rHGS cutoffs, we did not adopt these thresholds because they were developed in Western populations using different measurement modalities. The primary outcome was impaired physical performance at follow‐up, defined as a five‐time chair stand time ≥ 12 s [6]. Multivariable logistic regression models, adjusted for demographic, cognitive, functional and comorbidity covariates used in the original study, were applied to evaluate the associations of low MSS and low rHGS with performance decline. The study was approved by the Institutional Review Board of Taipei Veterans General Hospital (2018‐05‐003B), and all participants provided written informed consent. The study adhered to the 1964 Declaration of Helsinki and its later amendments.

In these supplemental analyses, low MSS remained significantly associated with impaired physical performance (adjusted OR 1.49, 95% CI 1.11–1.99; *p* = 0.008). Low rHGS normalized to body weight and BMI were also significantly associated with performance decline (OR 1.43 and OR 1.54, respectively), whereas rHGS normalized to height^2^ was not (Figure 1). The effect sizes for rHGS‐weight and rHGS‐BMI were comparable to MSS, although MSS remained the only metric consistently significant across all strength‐standardization approaches. By contrast, neither absolute grip strength nor the muscle quality index predicted performance deterioration. These findings indicate that both MSS and selected forms of rHGS capture functional vulnerability more effectively than absolute strength measures. Notably, MSS remained significantly associated with impaired performance even when body fat percentage was included in the models, underscoring its distinct utility in adiposity‐related functional impairment. This robustness supports the emerging role of MSS as a novel indicator for identifying sarcopenic obesity, which is characterized by preserved or elevated muscle mass but diminished contractile efficiency.

**FIGURE 1:**
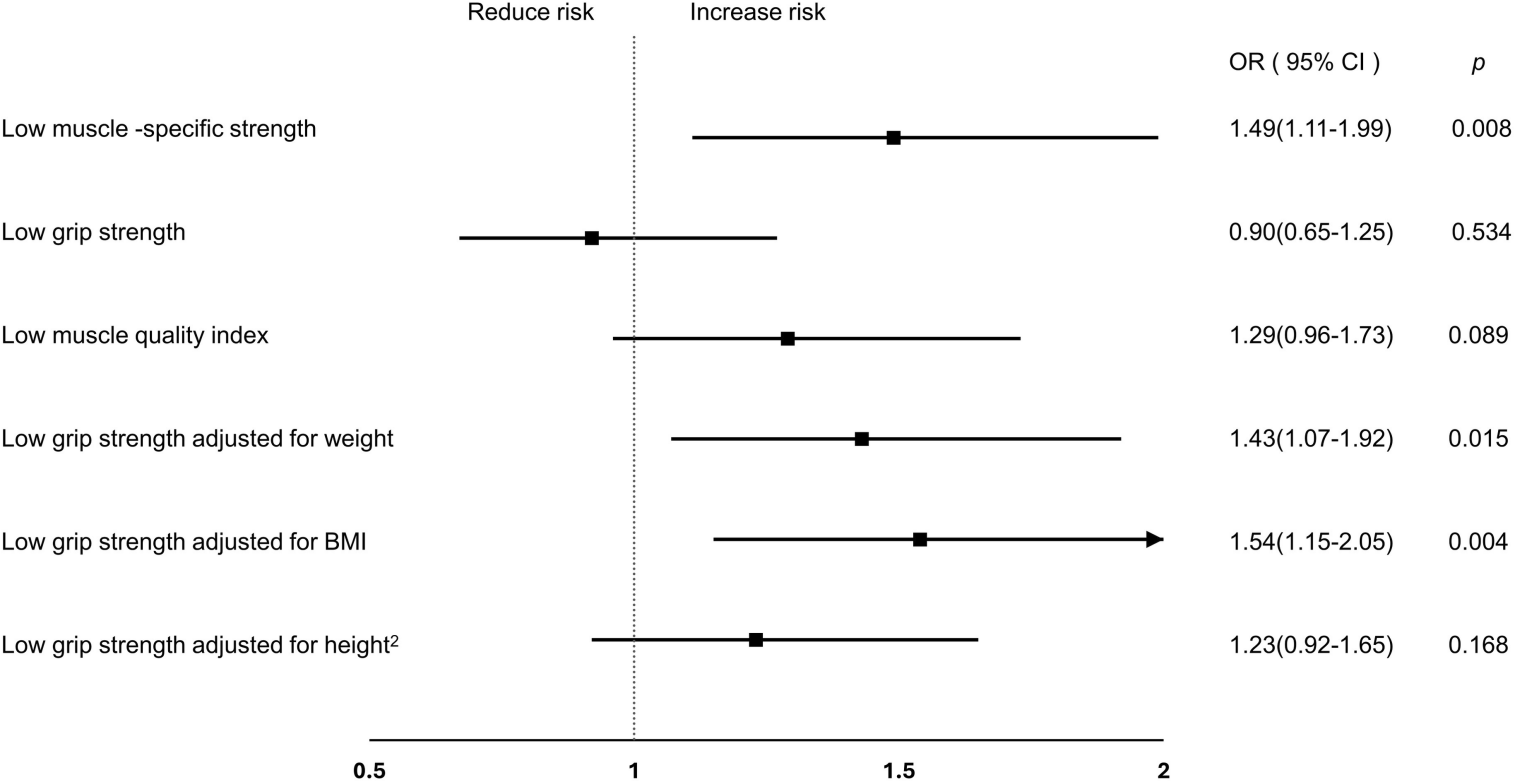
Grip strength adjusted for body size metrics to predict physical decline.

The AWGS 2025 and GLIS frameworks emphasize the need for functional metrics that reflect both quantity and quality of muscle [4, 5]. Our supplemental results align with these evolving perspectives. While rHGS reflects whole‐body gravitational load and metabolic burden, MSS quantifies the efficiency of force production per unit of active muscle tissue, offering a region‐specific assessment of neuromuscular quality. This distinction is particularly meaningful in individuals with higher adiposity, where muscle quantity may remain preserved yet contractile performance is impaired [7]. Importantly, our previous supplementary analyses demonstrated that MSS‐defined sarcopenic obesity was associated with significantly increased physical performance impairment [1], further supporting MSS as a sensitive and biologically relevant indicator in obesity‐related muscle dysfunction.

Beyond risk stratification, the distinct sensitivity of MSS to adiposity‐related impairments and neuromuscular inefficiency suggests potential applicability in multidomain interventions designed to promote healthy ageing. Because MSS captures contractile efficiency rather than muscle quantity alone, it may serve as a responsive and biologically meaningful indicator to monitor improvements in muscle quality, metabolic regulation, and functional resilience [8]. As multidomain interventions increasingly integrate exercise, nutrition and metabolic optimization strategies [9], MSS may serve as a valuable marker for detecting early functional gains, particularly in individuals with sarcopenic obesity, where conventional strength or mass‐based metrics may underestimate meaningful physiological improvement.

In summary, our supplemental analyses reinforce that both MSS and selected forms of rHGS predict physical performance decline, supporting the broader consensus that normalized strength metrics provide superior functional insight compared with absolute grip strength. Consistent with AWGS 2025 and GLIS guidance, MSS offers a unique index of contractile efficiency that remains robust even in the context of elevated adiposity and may enhance the detection of sarcopenic obesity. While rHGS represents a practical, scalable tool in clinical settings, MSS provides an anatomically and physiologically grounded assessment of neuromuscular function and may inform future refinements to sarcopenia and sarcopenic obesity definitions. Together, these complementary approaches underscore the multifaceted nature of muscle function and the importance of integrating both structural and functional parameters in evaluating functional vulnerability among older adults.

## Funding

The study was funded by the National Science and Technology Council (NSTC‐113‐2314‐B‐A49‐060 and NSTC 114‐2314‐B‐A49–042) and Taipei Veterans General Hospital (114 VACS‐001). It is important to note that the research funder played no role in the study design, data collection or analysis, manuscript preparation or the decision to publish. Authors would like to thank the Interdisciplinary Research Center for Healthy Longevity of National Yang Ming Chiao Tung University from The Featured Areas Research Center Program within the framework of the Higher Education Sprout Project by the Ministry of Education (MOE) in Taiwan for supporting the research work.

## Conflicts of Interest

The authors declare no conflicts of interest.
